# Design and fabrication of dual responsive lignin-based nanogel via “grafting from” atom transfer radical polymerization for curcumin loading and release

**DOI:** 10.1038/s41598-021-81393-3

**Published:** 2021-01-21

**Authors:** Ali Dinari, Mahdi Abdollahi, Majid Sadeghizadeh

**Affiliations:** 1 Polymer Reaction Engineering Department, Faculty of Chemical Engineering, Tarbiat Modares University, Tehran, Iran; 2 Department of Genetics, Faculty of Biological Sciences, Tarbiat Modares University, Tehran, Iran

**Keywords:** Biotechnology, Cancer, Chemical biology, Drug discovery

## Abstract

The story of human dreams about curing all diseases, disorders and lesions is as old as human history. In the frontier of medical science, nanomedicine is trying to solve the problem. In this study, inspired by nanotechnology and using “grafting from” approach, a novel lignin-based nanogel was synthesized using atom transfer radical polymerization (ATRP) method. *N*-isopropylacrylamide (NIPAM) and *N*,*N*-dimethylaminoethylmethacrylate (DMAEMA) comonomers were graft copolymerized from fully brominated lignin as ATRP macroinitiator to synthesize lignin-g-P(NIPAM-co-DMAEMA) nanogel (LNDNG). By controlling the initial comonomer compositions and ATRP conditions, four LNDNG systems with different lower critical solution temperatures (LCSTs) of 32, 34, 37 and 42 °C were prepared. The LNDNGs were evaluated by GPC, FT-IR, ^1^H NMR, UV–Vis, DLS, SEM and TEM analyses. The prepared nanogels exhibited an average diameter of 150 nm with dual temperature and pH responsiveness. Curcumin (CUR) loading capacity and encapsulation efficiency of the LNDNGs were 49.69% and 92.62% on average, respectively. The cumulative release amount of loaded CUR was observed to be 65.36% after 72 h. The new lignin-based NGs proposed in the present work seems to be a promising, safe and comparable system in a near future.

## Introduction

Diseases and lesions are the most challenging factors in dealing with human survival. From ancient world until now, researchers have tried to overcome the human health problems. In this respect, some traditional medicine scientists who believe in the medicine of immortality are trying to achieve a combination drug that cures all the human diseases and prolonged the life indefinitely^1–3^. Nowadays, in the frontier of science, nanomedicine is trying to realize these dreams^4^. Diagnosis and curing diseases via nanotechnology are among the most challenging new approaches. There are plenty state of the art innovations in the field of nanomedicine for healing diseases and lesions; e.g. all new drug delivery systems including micelles, inorganic particles, theranostic agents etc.^5^.

Nanogels as a new generation of drug delivery vehicles represent excellent loading, carrying and releasing patterns in their target sites. These carriers are the improved microscopic version of hydrogels, so that they may absorb by areas not accessible to hydrogels. This feature renders them an ideal candidate for delivery of drug agents inside the cells^6^. Nanogel is a nanoparticle composed of flexible three-dimensional polymeric network with a great capacity in drug loading^7^. Lignin as a second renewable biomaterial, after cellulose, and a gift from nature is available everywhere. Phenolic nature of this complex structure with multiple functional groups making it an excellent candidate to be applied in the industry, environmental science and lately in the medicine science^8^.

Other familiar synthetic polymers including poly(*N*-isopropyl acrylamide) (PNIPAM) and poly(*N*,*N*-dimethylaminoethyl methacrylate) (PDMAEMA) have an obvious history of applications. Temperature responsive behavior of the PNIPAM has been proved by numerous researches previously. It has been accepted today that the lower critical solution temperature (LCST) of pure PNIPAM is around 32 °C^9^. Phase transition of PNIPAM across the LCST as a reversible phenomenon are resulted from hydrogen bonding and hydration interaction below and above the LCST respectively. Hence, this potential ability gives a list of opportunities in industry and medicine. The PDMAEMA as the other synthetic polymer shows a temperature and pH responsive behavior. This polymer is a weak polybase with a LCST adjustable through the degree of protonation of its tertiary amine groups (pK_a_ = 7.4)^10^. However, dual functionality of the PDMAEMA works only within the pH range of 6–8^11^. Drug delivery systems advancement has introduced a great achievement in new age of nanomedicine where many new systems with favorable features are annually reported. However, probable side reactions of carrier systems have still remained. The features such as stimuli responsiveness, accuracy of loading and release processes, biocompatibility and biodegradability are involved in challenge. Besides to these features, physicochemical properties of drugs also play a critical role in carrier’s acceptance by medical society. For example, many drugs have hydrophobic nature (such as cancer drugs) and they are not easily accessed by cells. Hence, there is necessity to load them in a quite suitable carrier. To do this, design and fabrication of a proper carrier is the most important issue. Therefore, one should look for a carrier that has all the above-mentioned characteristics and performs its assigned function in the correct form considered in the theoretical studies.

In this research, a biodegradable and stimuli-responsive nanogel named as lignin-g-P(NIPAM-co-DMAEMA) nanogel (LNDNG)) was synthesized for the first time with the aim of drug delivery to cancer cells. Indeed, P(NIPAM-co-DMAEMA) copolymer chains were grafted from fully brominated lignin nanoparticle using atom transfer radical polymerization (ATRP). The high capacity of relatively hydrophobic lignin to absorb the curcumin (CUR), responsiveness of grafted copolymer including the PNIPAM (temperature responsiveness) and PDMAEMA (pH responsiveness), high loading capacity and sustained drug release behavior are remarkable features of this new nanogel (NG) system. To our knowledge, there is no published article about graft copolymerization of NIPAM and DMAEMA from lignin biopolymer. Moreover, due to existence of a relatively hydrophobic nature of the lignin in the NG, enhanced loading of hydrophobic drugs with poor water solubility was expected. All the important parameters of this nanogel system such as synthesis, modification, drug loading and release profile and finally its cytotoxicity were studied using suitable analytical techniques.

## Results and discussion

**Figure 1:**
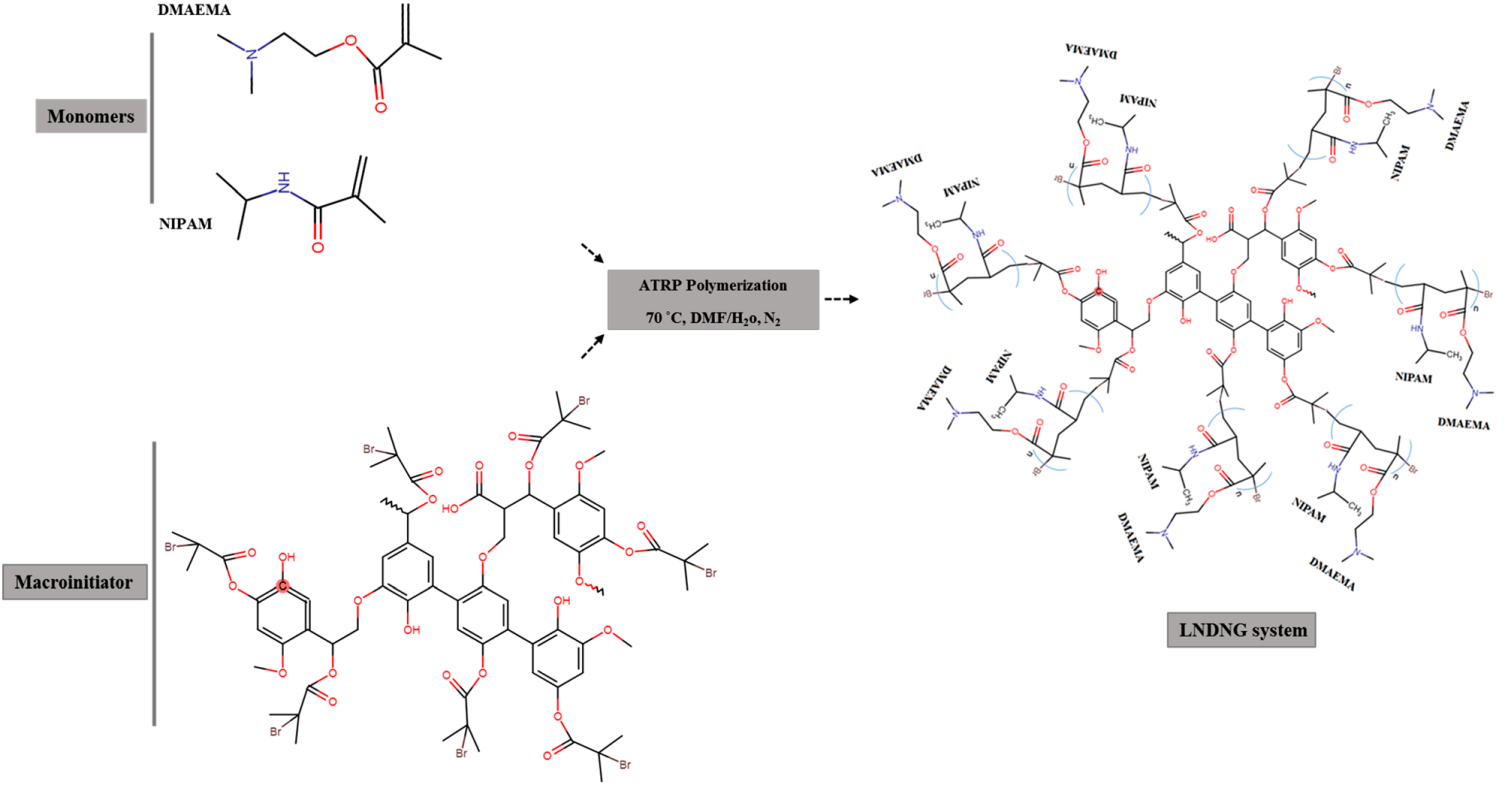
Schematic representation of fabrication procedure of lignin-g-P(NIPAM-co-DMAEMA) nanogel (LNDNG) via “grafting from” ATRP approach.

Lignin and its opportunities show a new age of biopolymer application growing in humans’ life. Among all the scientific published patents and papers, industrial applications of lignin are the most and medical ones are the least^12^. Lignin-based materials in combination with the other polymers (synthetic or natural) have presented a variety of application^13^. However, amorphous nature, heterogeneity and complexity of the lignin structure (due to polymerization of the various alcohols and their methoxylations) impeded its application in the high value products. The conversion of lignin from mass state to nanostructures may overcome such limitations and improve the properties of lignin such as antioxidant activity, biocompatibility and biodegradability^14^. It is well known that drug delivery systems are based on the nanoparticle systems such as inorganic nanoparticles, liposomes, micelles and so on. However, stability, therapeutics loading capacity, responsiveness to stimuli factors, site—specific delivery, release management process and dissociation to their component parts are the issues of concern^15,16^. Despite all these issues, NGs are the promising candidate for drug-delivery approaches. In fact, NGs have significant applications in the fields of biosensing, bioimaging, tissue engineering and the excellent impact in the drug delivery area. These colloidal stable particles have small size, high stability, high loading capacity, large surface area to manipulation with bioactive molecules^17^. These intelligent carriers with flexible structures and nano-metric diameters have exhibited smart behavior to protect and release therapeutic agents^7^. It has been proven that flexible and soft structure of NGs can allow them easier penetration, prolonged time in circulatory system and taken up by cells more efficiently^18–20^. Furthermore, biodegradable NGs (among the classified into nondegradable and degradable ones) are more promising for biomedical applications. Due to manipulating their physical properties, functionalizing with targeting ligands and stimuli-responsive groups, they are able to act more intelligent in loading and releasing of the therapeutic agents in a spatial–temporal and degrading under cellular environments^21,22^. There are numerous articles (reviews and researches) which focused on NGs-based drug delivery. Improving the methods of NGs preparation, getting closer to more biodegradability (based on new compounds of natural and synthetic polymers) and triggering via stimuli (thermo, pH, and/or redox) act as driving forces^23–27^. In this research, LNDNG system was introduced for the first time (Fig. 1) and its potential properties in drug delivery were investigated. Drug loading capacity, biocompatibility, biodegradability and toxicity effects, stimuli response and drug release behavior were evaluated and recorded as the achievement of this study.

### Characterization of brominated lignin

Molecular weight of the fully brominated lignin as the macroinitiator in ATRP polymerization was measured using gel permeation chromatography (GPC). Number-average molecular weight ($\overline{M}_{n} )$ of modified lignin (fully bromination of all the hydroxyl groups, i.e. aliphatic and aromatic ones, by α-bromoisobutyryl bromide) was obtained to be 2146 g/mol (Table S1, supporting information) which was higher than that of unmodified lignin (about 1000 g/mol). It means that all hydroxyl groups have fully been brominated.

### Characterization of L-g-PNIPAM and L-g-PDMAEMA

Two homopolymers grafted onto lignin, i.e. L-g-PNIPAM and L-g-PDMAEMA, were synthesized in the absence of MBA crosslinker and then characterized. Their structures were analyzed using the FT-IR and ^1^H NMR spectroscopy. FT-IR spectra results (Fig. S1, supporting information) of synthesized lignin-g-homopolymer were same as those obtained for the NGs prepared in the presence of MBA as the crosslinker. Moreover, ^1^H NMR results (Fig. S2 for L-g-PNIPAM, supporting information) approved the presence of homopolymers grafted onto lignin from which molecular weight can be estimated. Based on the peak intensity in the ^1^H NMR spectrum, number-average molecular weight of L-g-PNIPAM was estimated to be 73,360 g/mol. This finding indicated that homopolymer chain, i.e. PNIPAM, grafted onto lignin have an appropriate length to cover the inner hydrophobic lignin.

### Synthesis and characterization of NG systems

ATRP polymerization of NIPAM and DMAEMA initiated from lignin (as macroinitiator) in the presence of MBA crosslinker has been reported previously. Mild conditions at room temperature and water as the solvent has been reported^28^. However, miscibility between H_2_O and DMF can persuade a particular interest to develop a wide range of copolymer synthesis. In the present study, constant ratio of H_2_O/DMF (3.5/1.5, v/v) and also constant molar ratios of monomers (with variable molar ratio of each monomer)/CuBr/PMDETA/Br in the macroinitiator ([NIPAM + DMAEMA]/[CuBr]/[PMDETA]/[Br] = 100:2:2:1) were used to obtain LNDNG systems with desirable properties. In order to attain well-controlled ATRP, ratio of H_2_O/DMF as the solvent, reaction volume and reaction time were controlled. In fact, existence of DMF enhances solubility of the lignin and inhibits unwanted reactions. Fabricated nanogel systems had its own specific LCST which were characterized by DLS and UV–Vis measurements. In Table 1, molar ratios of monomers in the initial feed, overall conversion and LCST of the produced nanogels have been given.

**Table 1:** Details of of lignin-based NGs synthesized in the present work.

	Lignin-g-PNIPAM	copolymer I	copolymer II	copolymer III	copolymer IV
NIPAM/DMAEMA in feed (mol/mol)	100/0	93.75/6.25	87.5/12.5	85/15	75/25
Conversion	58.90	51.10	71.81	74.58	57.94
LCST (°C)	32	32	34	37	42

### FT-IR analysis

**Figure 2:**
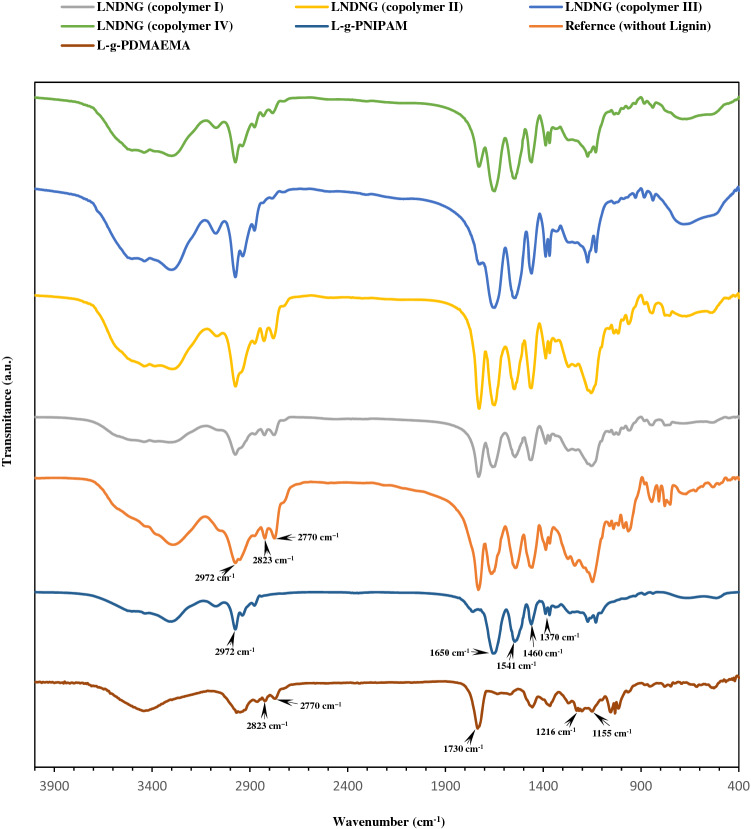
FT-IR spectra of various nanogel systems.

Nanogels were characterized by FT-IR spectroscopy as the first check point. Corresponding spectra are shown in Fig. 2. As shown in this figure, the characteristic peaks appeared at FT-IR spectra of nanogels were assigned to the corresponding functional groups, illustrating that nanogels have been prepared successfully. The peaks located at 1155 cm^−1^ and 1730 cm^−1^ were ascribed to the C–O–C ether linkage (stretching) and the C=O (stretching) of DMAEMA, respectively. The C=O stretching vibration of amide group also exists in the NIPAM backbone. peak appeared at 1650 cm^−1^ refers to amide carbonyl group of NIPAM monomer^29^. The above-mentioned peaks can be observed in the lignin graft copolymer samples. Peak appeared at 1620 cm^−1^ in the spectrum of NIPAM monomer or 1638 cm^−1^ in the spectrum of DMAEMA monomer can be ascribed to adsorption band of the C = C group^29,30^. Presence or absence of above-noted peaks at the resulting spectrum could be used to elucidate failure or success of the nanogels synthesis. In this respect, disappearance of the C = C groups peaks in the spectrum of samples indicated that they were successfully reacted in the ATRP polymerization. Characteristic peaks at wave numbers of 1541 cm^−1^, 1460 cm^−1^ and 1370 cm^−1^ can be ascribed to C-N (stretching), CH_2_ (bending) and CH_3_ (bending) groups of NIPAM respectively. It is noteworthy that groups of CH_2_ and CH_3_ are also present in the DMAEMA. However, their position at L-g-PDMAEMA spectrum were shifted to 1370 cm^−1^ and 1216 cm^−1^ respectively. The peaks appeared at 2770 cm^−1^ and 2823 cm^−1^ (shown in spectra of all resultant nanogels except for L-g-PNIPAM) were assigned to the C-H bond of the N(CH_3_)_2_ group of DMAEMA. The next valuable peak has been appeared at 2972 cm^−1^, relating to the C-CH_3_ group of NIPAM incorporated into the polymer chain. On the other hand, characterization of lignin functional groups, i.e. aromatic ring, methoxyl, hydroxyl (aliphatic and phenolic) and carboxylic groups has its own significance. In this respect, lignin aromatic ring has an indicator peak appeared at 1400 cm^−1^ to 1600 cm^−1^ in the FT-IR spectrum. Nevertheless, these peaks with low intensity have been overlapped by strong peaks of NIPAM and DMAEMA groups. The carbonyl groups of lignin carboxyl groups have also been appeared at around 1716 cm^−1^, but the same strong peaks related to NIPAM and DMAEMA would interfere with it. In addition, C–H stretching vibration (CH_2_ and CH_3_ groups) of methoxyl group and aliphatic groups of lignin appears at 2750 cm^−1^ to 3000 cm^−1^ in the FT-IR spectrum^31^. However, this may be unified with the same groups of NIPAM and DMAEMA as well. Eventually, a broad peak located at 3200 cm^−1^ to 3400 cm^−1^ could be ascribed to hydroxyl group of carboxylic group in the lignin. It should be noted that lignin’s functional groups had been characterized in the previous research^32^.

### UV–visible spectroscopy

**Figure 3:**
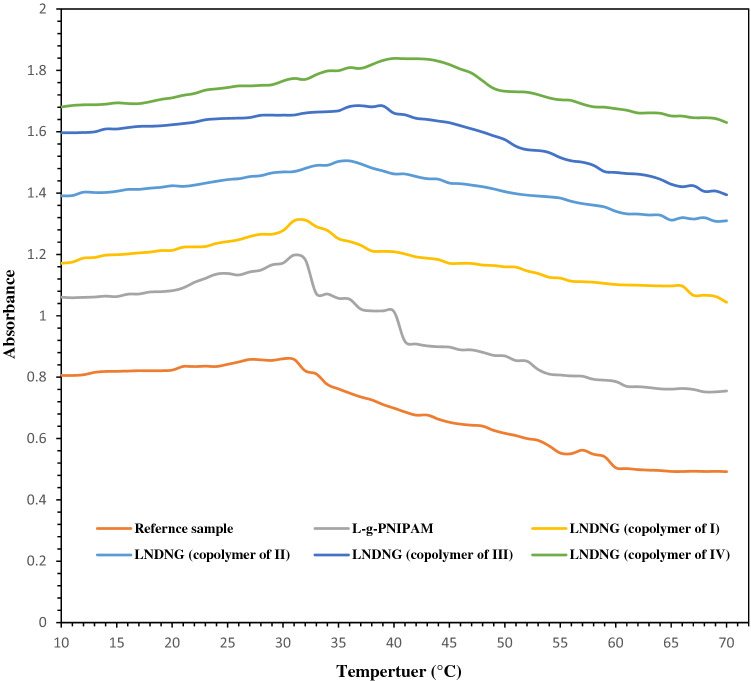
LCST determination of nanogel systems using UV–Visible spectroscopy via recording absorbance versus temperature of nanogel samples by heating rate of 1 °C/min at 500 nm.

To determine LCST behavior of thermoresponsive polymers, different techniques such as calorimetry (based on differential scanning calorimetry (DSC)), ^1^H NMR spectroscopy, turbidimetry and dynamic light scattering (DLS) are always used. Many researches have used micro-DSC as the common and reliable device to characterize thermal behavior of polymers^33^. ^1^H NMR is another valuable instrument for LCST evaluation. Nevertheless, cross-linked network of nanogels (as a case in the present study) prevents ^1^H NMR usage^34^. On the other hand, UV–Visible spectroscopy (turbidimetry) and DLS are the robust methods recommended for determination of the LCST phase transition^35^. Here, turbidimetry was applied to determine the LCST behavior of NG solution in elevating temperature ranges from 10 to 70 °C. Results are shown in Fig. 3. Indeed, the transparent aqueous solution observed at temperatures blow the LCST can be attributed to strong hydrogen bonds between the polymer chains and surrounding water, resulting in the hydrophilic NGs. By rising temperature to LCST and above it, polymer globules started to appear through the substitution of polymer-water hydrogen bonds by polymer–polymer interactions. It is clear that transition from hydrophilic coil to hydrophobic globule results in the shrinkage of polymer chains, leading to change of the NGs configuration above their LCST^36^. Truly, fabricated nanogels with particular LCST were achieved via changing molar ratios of two monomers (i.e. NIPAM and DMAEMA) in the initial feed and controlling condition (i.e. reaction time and thereby conversion) of ATRP. All the appeared curves in Fig. 3 correspond to the cloud point (LCST) at defined temperatures, confirming accuracy of the experiments. Replacement of NIPAM by DMAEMA could change the balances of hydrophobic-hydrophilic interactions in copolymer backbone and consequently, critical point temperature of LCST will be changed. LCST results obtained in the present wok were in a good agreement with the previous studies^37^. Temperature range of LCST process along the elevating temperature could prepare valuable information about LCST phenomenon of the NGs. In fact, LCST curves have differences in starting, duration and plateau phases. In this case, each NG compound indicates a specific characteristic. For example, LNDNG systems generated from copolymers of III or IV exhibited the LCST transition in a distinctive temperature range, different to others. The resultant curves shown that phase transitions of NG compounds have occurred at temperatures around predicted temperature ranges. These findings indicated that each NG sample has presented its own defined LCST. LCST of 32 °C was observed for three NG systems including L-g-PNIPAM, reference sample and LNDNG (I). On the other hand, the LNDNG systems II, III and IV showed distinctive LCST at 34, 37, 42 °C, respectively. In fact, different LCST behavior is originated from different DMAEMA amount incorporated into copolymer compounds. In other words, lowest amount of DMAEMA monomer incorporated into the first two NG compounds (except L-g-PNIPAM which has no DMAEMA monomer in the structure), while DMAEMA amount incorporated into the copolymer chains increased in second three LNDNG compounds, resulting in the raised LCST systematically. Therefore, gradual and systematic change in LCST of NG compounds would be a good feature to control release of the drugs. Hence, biomedical application of thermoresponsive polymers via point view of the LCST transition effects has been described in details^35^.

### DLS analysis

**Figure 4:**
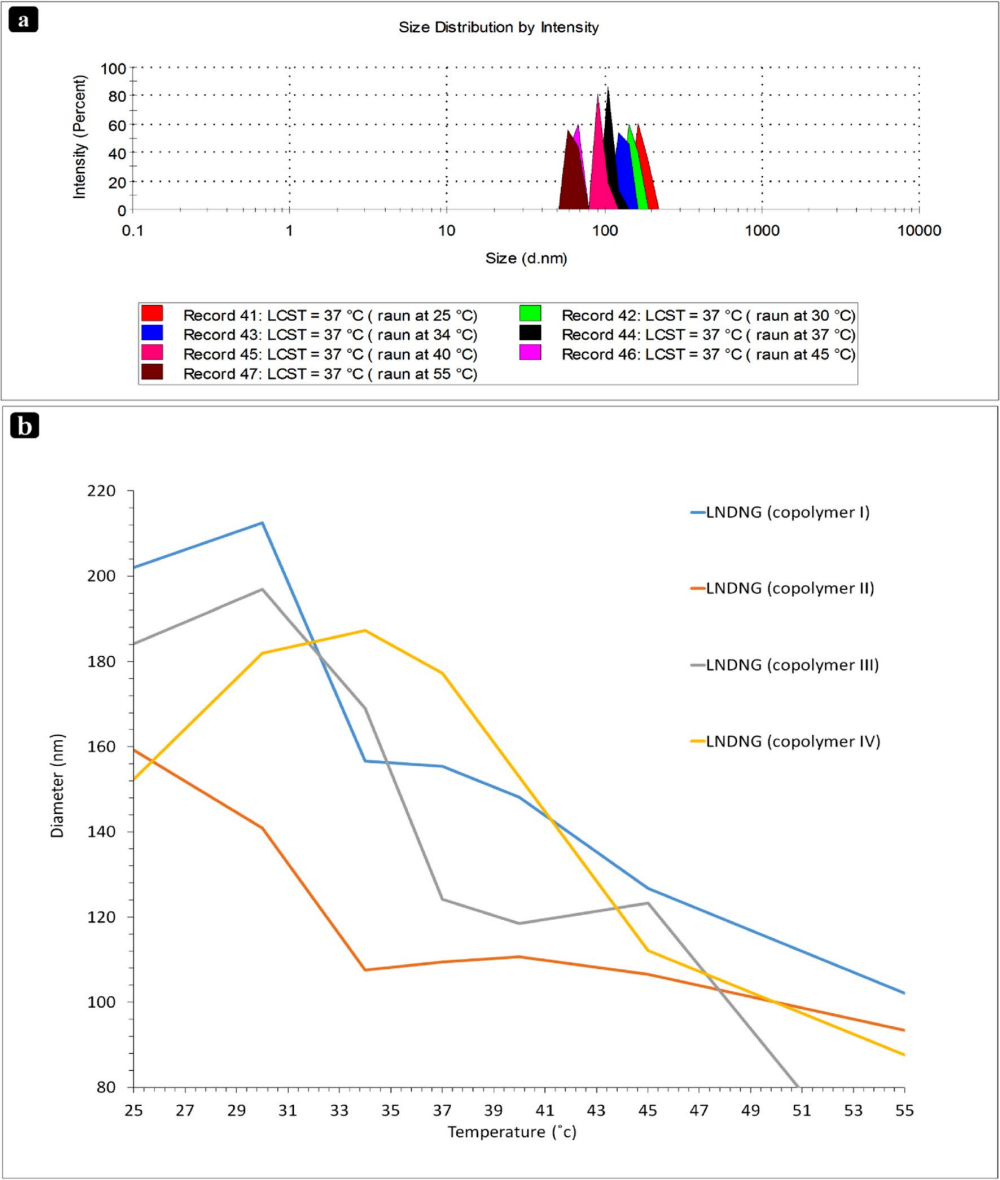
DLS results of nanogel systems: (**a**) nanogel particle size along with elevating temperature (from right to left, seven defined points of temperatures) and (**b**) diameter versus temperature of LNDNG systems.

DLS analysis was also performed to investigate LCST behavior of NGs with more detail as well as to estimate particle size and zeta potential of NGs (Fig. 4 and Table 2). Findings revealed difference LCST of NGs which were in a good agreement with UV–Visible findings. Four critical points of LCST occurrence (i.e. 32, 34, 37 and 42 °C) are related to four different compositions of the copolymers in the LNDNG systems (i.e. I, II, III and IV, respectively). The pH of NGs was kept constant at pH = 7.4 to investigate effect of only temperature on the NGs behavior.

**Table 2:** samples details related to LCST behavior investigation under seven defined points of temperatures.

Temperatures	Runs	Copolymer I ( LCST = 32 °C)			Copolymer II (LCST = 34 °C)		
		Duration used (s)	Attenuator	Count rate (kcps)	Duration used (s)	attenuator	Count Rate (kcps)
25 °C	Run 1	60	10	202.4	60	8	287.0
	Run 2	60	10	223.8	60	8	287.0
30 °C	Run 1	60	11	488.0	60	8	292.0
	Run 2	60	11	577.7	60	8	298.3
34 °C	Run 1	60	10	329.1	60	8	315.3
	Run 2	60	10	341.9	60	8	302.3
37 °C	Run 1	60	10	272.2	60	8	295.0
	Run 2	60	10	257.8	60	8	292.3
40 °C	Run 1	80	10	253.0	60	8	310.6
	Run 2	80	10	257.4	60	8	307.8
45 °C	Run 1	70	10	219.5	60	8	295.0
	Run 2	70	10	198.3	60	8	296.5
55 °C	Run 1	70	10	152.0	60	8	278.1
	Run 2	70	10	169.5	60	8	290.3

		Copolymer III (LCST = 37 °C)			Copolymer IV (LCST = 42 °C)		
		Duration used (s)	attenuator	Count Rate (kcps)	Duration used (s)	attenuator	Count Rate (kcps)
25 °C	Run 1	60	8	234.2	60	8	239.4
	Run 2	60	8	244.8	60	8	227.6
30 °C	Run 1	70	8	224.2	70	8	248.6
	Run 2	70	8	227.2	70	8	242.3
34 °C	Run 1	80	8	198.7	60	8	222.8
	Run 2	80	8	218.1	60	8	223.5
37 °C	Run 1	70	8	209.6	70	8	199.2
	Run 2	70	8	223.6	70	8	201.2
40 °C	Run 1	60	8	195.9	70	8	210.1
	Run 2	60	8	201.9	70	8	196.8
45 °C	Run 1	60	8	241.0	80	8	195.3
	Run 2	60	8	226.4	80	8	195.7
55 °C	Run 1	70	8	243.8	70	8	210.3
	Run 2	70	8	207.3	70	8	211.5

As mentioned before, shrinkage of the copolymers chains and transition from hydrophilic coil to hydrophobic globule starts at LCST point. Consequently, at this point, LNDNG particles start to become smaller in their size (Table 3). Effect of LCST on the NG configuration and its ideal response to temperature stimulus is obvious. Similar results about LCST investigation using the DLS technique have been published by others^38^. The other interesting issue is behavior of PDMAEMA at pH range of 6–8. At lower pH, NG of L-g-PDMAEMA showed the particles with larger dimensions in comparison with those at higher pH. It can be attributed to the PDMAEMA nature. Truly, tertiary amine groups of PDMAEMA chains in the lignin-based NGs are protonated in lower pH, resulting in the extended chains with larger dimensions, while by increasing pH, protonated chains convert to neutral ones, resulting in the collapsed chains with smaller dimensions. Similar results has been reported for PDMAEMA in the literature^39^. In addition, average diameter of L-g-NIPAM and L-g-DMAEMA NGs indicated that L-g-PNIPAM NGs have smaller size although it changes by elevating the temperature (due to LCST transition). Also, polydispersity index (PDI) values of particle size increased at temperature higher than LCST. It may be attributed to created hydrophobic forces and shrinkage of PNIPAM chains above their LCST. The tendency of L-g-PNIPAM NGs towards aggregation lead to larger and polydisperse particles.

**Table 3:** Result of DLS data indicating the reduction of nanogel particle size (nm) by increase in temperature.

LNDNG systems	Temperatures (°C)						
	25 °C	30 °C	34 °C	37 °C	40 °C	45 °C	55 °C
Copolymer I ( LCST ≈ 32 °C)	202.03	212.5	156.6	155.4	148.2	126.7	102.1
Copolymer II (LCST ≈ 34 °C)	159.2	140.8	107.5	109.4	110.6	106.5	93.36
Copolymer III (LCST ≈ 37 °C)	184.1	196.9	168.9	124.2	118.4	123.3	49.3
Copolymer IV (LCST ≈ 42 °C)	152.4	181.9	187.2	177.2	152.9	112.1	87.6

Four LNDNG contain four different copolymer compounds which have their own distinctive LCST behavior across the temperature rising.

Drug-free LNDNG was also monitored using DLS analysis with a more detail (Table S2, supporting information). Size distribution analysis showed that average particle size is smaller than 150 nm at fully extended state (i.e. at 25 °C) and reduces to a value lower than 100 nm at the shrinkage state (i.e. at above LCST). PDI values of all samples at temperature of 25 °C were lower than 0.2, indicating narrow particle size distribution. Above LCST, however; PDI values increased. The Z-average parameter as additional information data is useful to analyze LCST of NGs. Smaller Z-average values were recorded at temperature of 25 °C and its value increased at temperature of LCST. Significant change of Z-average data by rising temperature can be attributed to aggregated LNDNG at LCST point. Therefore, in addition to the PDI, the Z-average data also confirmed the above-mentioned events. It is noteworthy that LNDNG fate in cellular environments was totally different and disintegrated by cell compartments; hence, agglomeration of system is prevented. There is studies about biocompatibility and biodegradation of lignin-based nanostructures^40^. Behavior of drug-loaded NGs was the same as the drug-free ones; however, their particle size was slightly smaller than the drug-free ones. The observed difference may be due to partial condensation of NGs core (originated from double hydrophobic force created by CUR and lignin). This event could reform NG network into smaller sizes.

Zeta potential was monitored to indicate surface charge of NGs from which stability and their uptake by treated cells can be evaluated^41^. The nanogels were analyzed and results revealed a wide range of zeta potential values (Table S2, supporting information). For instance, reference sample (lignin-free sample) possess high values of positive zeta potential (26.8 mV), while LNDNG systems showed negative values. Moreover, drug-loaded and drug-free LNDNGs exhibited a little difference in the zeta potential values. For example, the drug-free and drug-loaded LNDNG2 showed negative zeta potential of −13.7 mV and − 32.1 mV, respectively, under basic condition. In addition, the surface charge of NGs were changed with pH. For example, acidic pH raised the zeta potential values in comparison to the basic pH. These observations can be related to DMAEMA role and synergistic effect of the lignin and CUR. Acidic condition could induce protonation of DMAEMA and consequently, surface charge of NGs shifts towards positive values. However, this may be vanished by increasing the pH range. Moreover, at non-acidic condition, the effective factor is referred to synergetic effects of lignin and CUR. Functional groups of CUR (hydroxyl and carbonyl) and lignin (carbonyl and more especially carboxylic acid) could increase negative surface charge of LNDNG systems. Previous literatures have described the efficacy of zeta potential on the drug delivery system. Cellular uptake and the fate of nanoparticles with different surface charges have been discussed^42,43^. Results obtained in the present study were confirmed by previous researches where values of zeta potential are consistent with the previous reports.

### EM imaging

**Figure 5:**
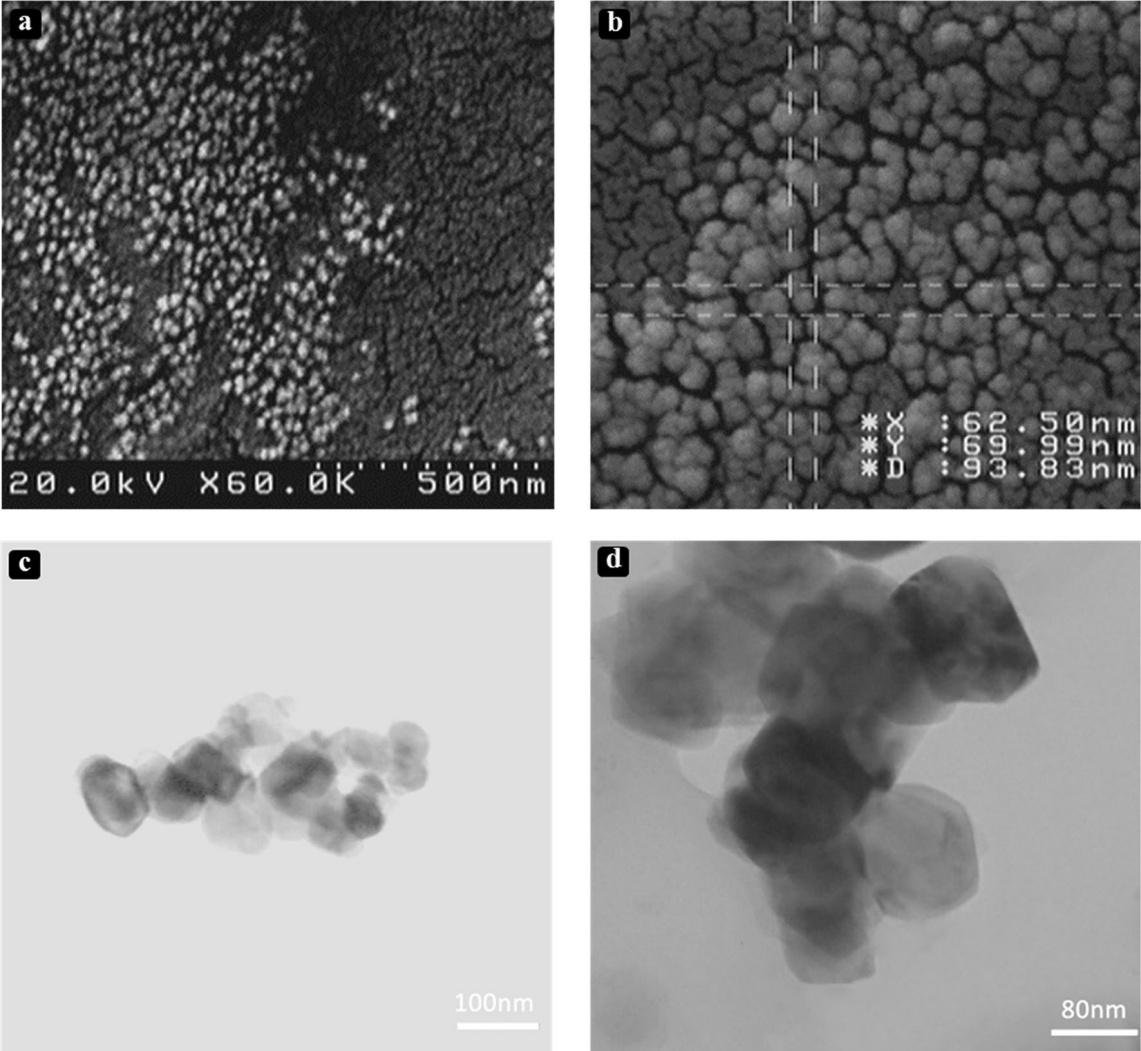
EM imaging of CUR-loaded LNDNG. Images of (**a**) and (**b**) refer to SEM micrographs and those of (**c**) and (**d**) belong to TEM micrographs.

Nowadays, morphological analysis of nanostructures via EM is an appropriate and recommendable method. In order to observe morphology of the produced NG compounds, TEM and SEM micrograph images were used. Morphological features of NG systems are showed in Fig. 5. Sizes and shapes of NGs at two states (empty and CUR-loaded) can be evaluated from this figure. Generally speaking, micrographs of NGs indicated that their average sizes at dry state are about 100 nm which is smaller than those at the hydrate state.

### Drug loading (DL) and encapsulation efficiency (EE)

The LNDNG capacity for DL content and EE was investigated and recorded. There is various nanostructure systems which are applied for drug delivery and shows a variety of pros and cons^44^. Todays, NG -based systems have been developed more and more and evaluated in the biomedical application^45,46^. The high capacity of NGs, for example, exhibits great potential to load more amount of drugs^47^. In fact, NGs are belong to a group of carriers which responses to high content of drugs^6^. Previous research has indicated that, as the amount of drug added to reaction mixture increases, the efficiency of drug-loaded in the NG decreases^48^. However, LNDNG system showed a negligible reduction of drug-loading efficiency (Table 4). According to these results, there was no significant reduction in the loading content and efficiency rate at higher amount of CUR drug. Actually, physicochemical properties of relatively hydrophobic lignin as the core of NG system renders a sufficient space capacity to absorb a great amount of hydrophobic CUR drug. It has been reported that incorporating drugs into carrier systems is controlled via their physicochemical properties and inner space shape^49^. High level of loading content and encapsulation efficiency were remarkable at the LNDNG system. DL and EE was obtained to be 49.69% and 92.62%, respectively, on average for all LNDNG systems. Nevertheless, it is clear from Table 4 that the above-mentioned parameters for reference lignin-free sample (DL = 1.55% and EE = 23.64%) and L-g-PNIPAM NG (DL = 4.34% and EE = 46.19%) are significantly lower. The distinct performance of the LNDNG systems compared to other NGs can be attributed to their composition. Presence of lignin improved potential loading capacity of LNDNG, resulting in more amount of CUR to be absorbed. There is similar reports on the excellent properties of lignin to absorb hydrophobic drugs^50^. Hydrophobic nature of both materials (lignin and CUR) act as pivotal mechanism to absorb CUR on the lignin macromolecule^51,52^. A case study on oral delivery of CUR via lignin nanoparticles has shown high affinity of these nanoparticles to entrap CUR molecules and record effective rate of EE^50^. Hyperbranched nature of the lignin make it as a proper candidate to absorb and carry hydrophobic and hydrophilic drugs^53^. Via acting as a drug store cavity, presence of lignin in the LNDNG systems enhances drug loading ability. It should be mentioned that cross-linked network of P(NIPAM-co-DMAEMA) grafted onto the lignin prevents undesirable dissolution of the absorbed CUR. The P(NIPAM-co-DMAEMA)-based nanoparticles have evaluated by previous studies and exhibited the acceptable evidences on DL and EE^54^. A soft-hydrogel system has been fabricated via blending the block copolymer of PDMAEMA-b-PNIPAM with polyvinyl alcohol (PVA). This research findings represented valuable information about hydrogel and its responses to pH and temperature^55^. The responsiveness of the carrier to stimuli factors can effect on its DL and EE.

**Table 4:** The results obtained on CUR-loading content and encapsulation efficiency by various NGs.

Part A																
Drug: carrier ratio	LNDNG I				LNDNG II				LNDNG III				LNDNG IV			
	acidic		basic		acidic		basic		acidic		basic		acidic		basic	
	DL	EE	DL	EE	DL	EE	DL	EE	DL	EE	DL	EE	DL	EE	DL	EE
1:100	5.035	72.61	62.17	98.79	16.01	76.21	33.34	94.91	39.21	97.61	34.20	94.90	58.48	98.62	15.50	84.97
2.5:100	15.29	90.02	9.152	83.43	13.03	72.27	32.92	86.81	14.14	73.87	20.76	80.59	14.56	77.36	35.57	86.35
5:100	15.77	90.35	6.757	78.37	30.26	85.82	16.46	76.70	24.72	96.14	1.546	23.62	61.46	95.70	5.413	51.98
7.5:100	4.339	69.40	15.52	90.18	44.48	89.89	17.60	77.87	13.55	73.04	18.33	78.57	58.19	95.29	19.07	79.23
10:100	5.806	75.50	13.46	88.60	59.04	92.19	23.69	82.57	39.96	96.55	62.08	92.54	55.89	93.81	20.65	80.51
15:100	17.33	91.29	9.031	83.23	58.14	92.08	29.42	85.47	61.62	96.99	66.92	93.89	54.23	94.96	15.24	75.30
25:100	18.41	91.86	11.21	86.32	51.56	91.16	36.87	88.05	65.34	94.75	65.34	94.75	50.62	94.16	52.67	91.33
35:100	15.26	90.00	18.02	91.66	53.80	91.49	39.59	88.78	61.08	93.42	64.23	93.68	61.13	92.44	53.09	91.39
50:100	15.83	90.39	19.63	92.43	60.41	92.35	45.66	90.13	62.62	94.88	47.93	90.55	59.75	92.27	47.37	93.93
100:100	13.97	89.04	24.17	94.09	40.42	88.99	70.00	93.33	55.92	91.79	37.03	88.10	49.07	90.75	56.02	94.50
Part B																

	Reference nanogel		Nanogel of L-g-PNIPAM													
	DL	EE	DL	EE												
5:100	1.87	27.28	4.33	48.06												
25:100	1.67	25.11	5.60	52.30												
100:100	1.13	18.54	3.11	38.22												

### Drug release process

**Figure 6:**
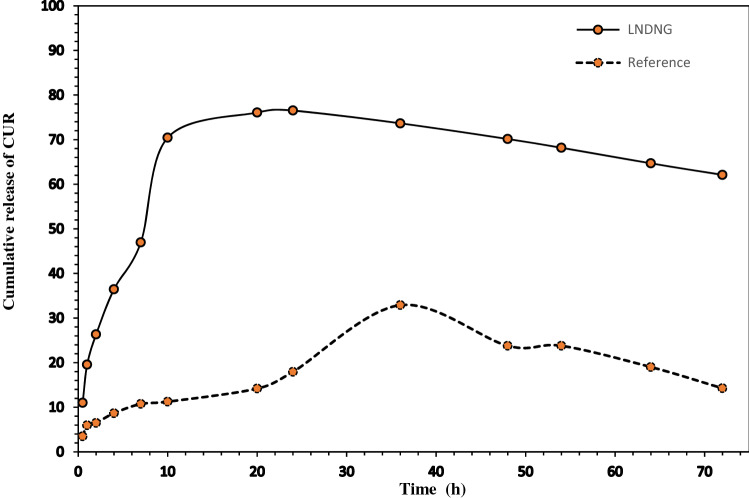
Comparison of CRU release behavioral pattern between LNDNG system and reference sample as a function of time.

Profile of drug release is an important basic parameter which has a great impact on the fate of drug carrier systems. In this research, drug release profile of LNDNG systems were investigated in response to temperatures including LCST and basic temperature (10 °C) and two pH values of 6.2 and 7.4 (Fig. 6). It is obvious that presence of lignin in NG systems shows a unique behavior in drug release process. Delay in release process and its effects on CUR diffusion in cellular environments is significant advantage of hydrophobic lignin. On the other hand, the reference sample (lignin-free) was not able to show such pattern. In fact, the rate and time duration of drug release were different between the LNDNG systems and the reference sample (Fig. 6). For LNDNG systems under study, the highest rate of drug release was occurred 10 h after beginning the release process. At this time interval and the next times (i.e. 16, 24, 36 and 48 h), the process of release showed smooth and steady state behavior with a minimum fluctuation in drug release. Comparison of upper and lower limits of temperatures across the LCST indicate a relative advantage in upper limit. This drug release process was in a good agreement with the previous researches^54^.

On the other hand, drug release profile at 10 °C (basic temperature) showed different pattern in starting point (earlier time), rate of release (lower) and more fluctuation in drug dissociation (Fig. S4, supporting information). It seems that the process of drug release profile at the temperature of 10 °C is induced by pH variation. This phenomenon can be related to the protonation/deprotonation of DMAEMA units. As mentioned before, DMAEMA monomers incorporated into the NG compounds exhibited a pH response behavior. Protonation of DMAEMA is responsible for the degradation and drug diffusion of the NG systems. It is clear that pH-responsive polymeric carriers utilize the mechanism of protonation/deprotonation as a common approach to exhibit their functions^5^. The DMAEMA units contain the tertiary amine functional groups which are deprotonated at basic pH and protonated at acidic pH^56^. These pH-dependent reactions are the major mechanism of drug release of the NG systems at temperature of 10 °C. Moreover, burst release behavior and fluctuating pattern of the drug release have been appeared for NGs at only this temperature. However, at temperatures equal to or higher than the LCST, shrinkage property of NIPAM units in combination with the pH responsivity of the DMAEMA units can inhibit undesired fluctuations. Incorporation of lignin (with tendency towards the CUR absorption) with P(NIPAM-co-DMAEMA) in the LNDNG systems can promote the sustained release of the absorbed CUR. Moreover, three dimensional networks of P(NIPAM-co-DMAEMA) chains along with bio-based hyperbranched lignin polymer may result in the fabrication of a nanogel with potential ability to absorb and encapsulate the hydrophobic drugs^57^. Drug-release profile of LNDNG systems showed a same pattern at tow defined pH values of 7.4 and 6.2. However, a relative supremacy in the sustained release and higher rate of the release were observed at pH of 7.4 (Fig. S5, supporting information). This may be attributed to the CUR release retardation in acidic condition. Indeed, hydrophobic nature of the lignin causes a resistance against the quick elution in harsh acidic pH such stomach ambience (pH 1.0–2.5)^50,58^. The average cumulative release of the CUR from the LNDNG systems reached 65.36% after 72 h. The recorded value for the reference sample was equal to only 11.23% in the same time period. It should be mentioned that the CUR release from the LNDNG systems is still continuing after 72 h. LNDNG systems have privileges for drug release. The first one refers to tendency of lignin core to absorb the CUR, and its low solubility in harsh acidic condition. The second one may be attributed to temperature-dependent phase behavior of PNIPAM where hydrophobic interaction at temperature equal to or higher than LCST increases, resulting in the shrinkage of the nanogel systems. The third one is originated from PDMAEMA and its effects on the drug release. Mechanism of protonation/deprotonation and responsivity to both stimuli (pH and thermal) will influenced overall reaction of the LNDNG systems. Delay in release process may be referred to the features of hydrophobic nature and low dissociation in acidic condition which are common in both lignin and CUR. As previously reported, hydrophilic/hydrophobic balance of lignin is related to hydroxyl (phenolic and aliphatic), carboxylic, methoxyl and aromatic rings as the functional groups^59^. It bears in mind that carboxyl groups have a pK_a_ about 5 and fully ionized at alkaline condition^60,61^. Mechanisms of combined action of all the mentioned factors determine degree of solubility and ionization of lignin by rising the pH range^62^. It seems that physicochemical properties of lignin display a critical role in controlling the rate and duration of CUR release. However, observations of burst release at temperature of 10 °C may be due to weak function of the LNDNG systems under this adverse condition. In this situation, release process has been affected by the pH as the only affecting factor. It has been reported that long-term sustained drug release could be resulted from the LCST’s temperature of NG systems with a suitable cross-linked networks. However, burst and faster release will be happened for systems that does not show LCST behavior^63,64^. To deliver drugs with sustained release, the LNDNG systems with their own unique properties seems to be an appropriate nanostructure candidate.

### MTT assays

**Figure 7:**
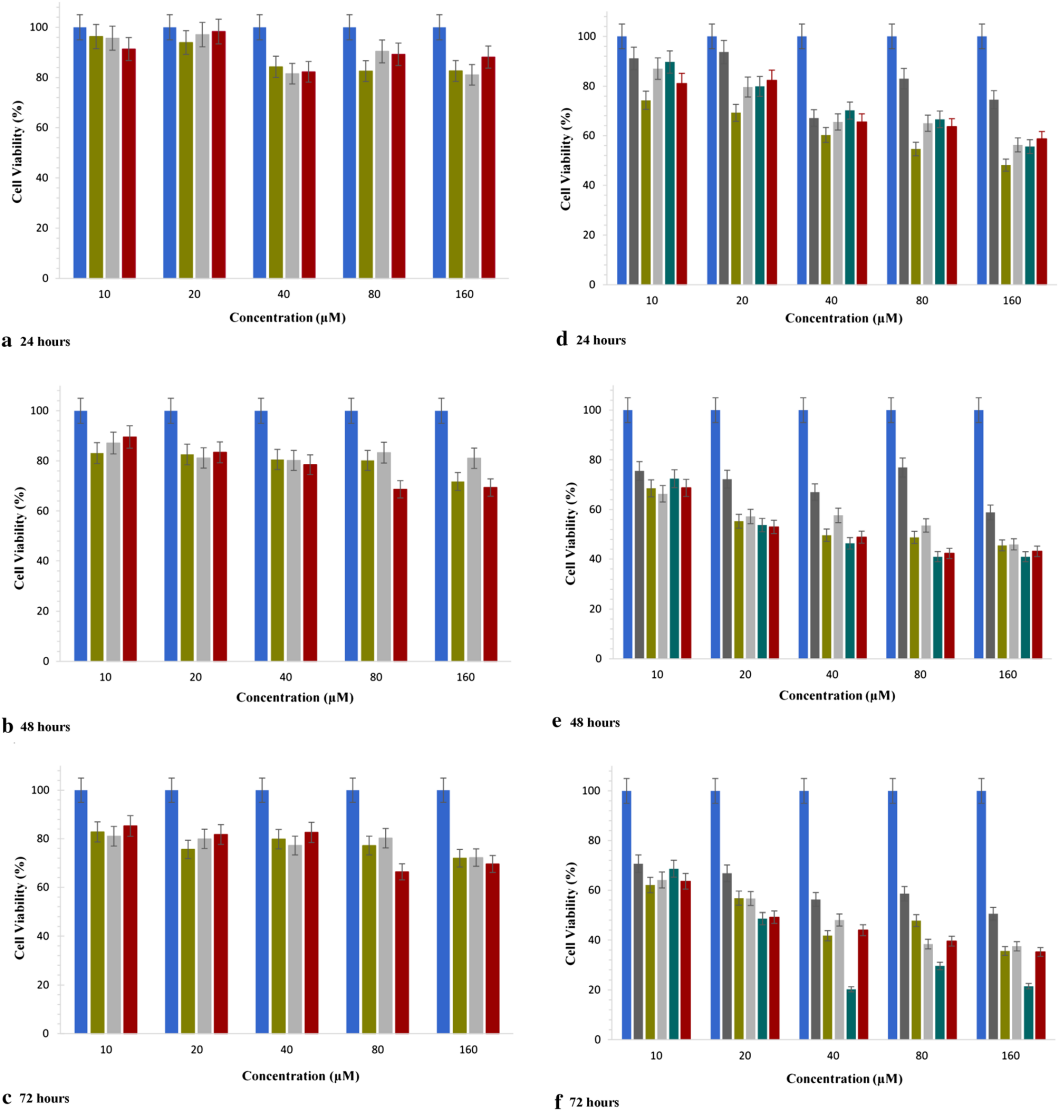
Treatment of hella cell with drug-free and CUR-loaded NG systems. Different colors were according to control (blue), free CUR (dark grey), reference sample (green), L-g-PNIPAM (gray), LNDNG number 3 (turquoise blue), and LNDNG number 2 (brown) respectively. Safety of drug-free samples (**A**–**C**) on viability of exposed cells were evaluated at three points of time (24, 48, and 72 h). Cytotoxic effects of CUR-loaded nanogel systems (**D**–**F**) on exposed cells were also represented.

As mentioned in the previous studies, MTT assay is just a simple conversion of tetrazolium salt to formazan via mitochondrial enzyme. Outcome of this action could be used to verify the pharmaceutical agents’ safety or toxicity on cell survival^65^. The results of MTT assay are shown in Fig. 7. Lignin, PNIPAM and PDMAEMA have shown no toxic effects on cell viability^54,66–68^. To evaluate the safety of the LNDNS systems, the CUR-free and CUR-loaded NG carriers, and free CUR were treated on the hella cell line. Results are shown in Fig. 7. According to this figure, cytotoxicity and cell damage affected by the drug-free LNDNG systems exhibited no symptoms. Indeed, safety of CUR-free LNDNG samples were guaranteed by monitoring viability of exposed cells at three points of time (24, 48, and 72 h). Observations showed that at low doses of drug-free NG systems, there was no an adverse effect on cells viability. The only negligible cell death was observed at high doses of drug treatments. However, treatments of drug-loaded nanogel systems showed different behavior. In this case, the minimum cell death rate may be attributed to the free CUR treated samples. In fact, at first 24 h, the most effective and the less effective nanogel systems were the lignin-free reference sample and the LNDNG ones respectivly. However, at times of 48 and 72 h, cell death rate of the LNDNG treatments increased and exceeded that of the reference sample. The CUR-loaded LNDNG systems demonstrated the maximum cytotoxicity by passing of time and released the CUR in a sustained manner.

## Material and methods

### Materials

*N*-isopropylacrylamide (NIPAM), *N*,*N*-dimethylaminoethyl methacrylate (DMAEMA) and *N*,*N*’-methylenebisacrylamide (MBA) were purchased from Sigma. Lignin (as Kraft lignin) and brominated lignin (i.e. α-bromoisobutyrylated lignin) (BrL) were prepared in polymer reaction engineering lab from Faculty of Chemical Engineering (Tarbiat Modares University, Tehran, Iran) using a procedure reported in the literature^32^. All solvents were prepared as pure laboratory grade. Basic alumina (Al_2_O_3_) purchased from Sigma. *N*,*N*,*N*′,*N*′′,*N*′′-pentamethyldiethylenetriamine (PMDETA), Cu(I)Br (98%) were prepared from Sigma. It should be noted that PMDETA was purified by passing through a neutral alumina column before use. Cu(I)Br (98%) was purified by stirring in glacial acetic acid overnight, filtering, and washing with dry ethanol. Dimethylformamide (DMF), acetone, and n-hexane were prepared from Mojallali company (Tehran. Iran). Dialysis membrane with 6–8 kDa (cut-off) was purchased from SpectrumLabs Company (San Francisco, USA). Deionized water was used in all the experiments. Curcumin (CUR) was prepared from Nano Daroo Alborz company (Tehran, Iran). MTT powder was prepared from Sigma. The Hella cell line has also purchased from Pasteur Institute Iran cell bank (Tehran, Iran).

### Methods

#### Nanogel (NG) synthesis

The NG systems were prepared via the following protocol. For a typical reaction, monomers (e.g., 11 mmol of overall monomers with variable amounts for each monomer), MBA (3% w/w relative to overall monomers) and macroinitiator (e.g. 16.74 mg brominated lignin (BrL) containing 0.11 mmol Br functional group) were dissolved in solvent mixture (6.0 mL of deoxygenated H_2_O and DMF mixture (3.5/1.5, v/v) and transferred to a 20 mL medical vial and degassed via purging with nitrogen gas for at least 30 min. In the next step, Cu(I)Br (e.g., 0.22 mmol, 15.8 mg) and PMDETA (e.g., 0.22 mmol, 19.04 mg) were mixed with 2 mL of solvent (H_2_O/DMF, 3.5/1.5, v/v) in 10 mL medical vial, sealed and degassed for 15 min under nitrogen gas. After the Cu(I)-PMDETA complex was formed, freshly degassed Cu(I)-PMDETA stock was added to the final reaction. Molar ratios of ingredients [NIPAM + DMAEMA]/[CuBr]/[PDMETA]/[Br] was chosen to be 100:2:21, respectively. It should be mentioned that Cu(I)-PMDETA complex was added slowly (droplet mode) to the final reaction. The final degassed reaction mixture was allowed to proceed for 24 h under magnetic stirring at 70 °C. The reaction was stopped by cooling and exposure to air, after which centrifuged at 13,000 rpm for 15 min at the room temperature. The supernatant was removed and obtained precipitate was dissolved in the deionized water and transferred to a dialysis bag (6–8 kDa, cut-off) for 1 week. In this step, further purification was performed by dialysis against a diluted HCl solution in water, with a pH of about 5 (± 0.3) at room temperature (about 25 °C). The diluted aqueous solution of HCl was regularly changed throughout the purification process.

#### Characterization of nanogels

Characterization of nanogel systems synthesized in the present work were performed through the following techniques.

#### FT-IR spectroscopy

FT-IR spectrum of each sample was obtained via FT-IR (Perkin Elmer, Frontier, USA) spectrometer after mixing with KBr powder.

#### UV–Visible spectroscopy

To determine the LCST of nanogel solution, turbidity measurements were performed on a UV–Visible spectrophotometer (Perkin Elmer UV/VIS Lambda, USA) and at a wavelength of 500 nm. In fact, turbidimetry is one of the most widely used method for determining the LCST of thermoresponsive polymer solutions. The measurement can be performed on a simple UV–Vis spectrometer with temperature control^69^. Samples were dissolved in appropriate solvent (H_2_O/DMF) to obtain desired concentration (5 mg/mL). In the next step, each sample was placed to cuvette (equipped with a tiny magnetic bar on its bottom, to stirring and hampering any precipitation) and heated from 10 to 70 °C. The transmittance was measured during at least two controlled cooling/heating cycles with a cooling/heating rate of 1 °C/min. Temperature range of 10 °C to 70 °C was applied for heating the solution where the transmittance of light through the solution was constantly measured by spectrometer. The metastability assessment was performed by heating the samples to 70 °C followed by cooling to 10 °C (heating and cooling cycles with repetition of 2 times). All samples were analyzed in three replicated experiments.

### DLS analysis

LCST behavior of NG systems as well as particles’ size and zeta potential of NGs were evaluated by DLS measurements. To measure LCST, lyophilized powder of NG samples were dissolved in proper solvent (H_2_O/DMF) and purified via a Millipore filter (5 µm pore size). Then, NG dispersions with appropriate concentration (50—100 μg/mL) were prepared. In the next step, samples were transferred to the DLS cuvette and heated from 25 °C to 55 °C, (at 7 defined temperatures of 25, 30, 34, 37, 40, 45 and 55 °C). At each temperature, the samples were equilibrated for 600 s, and then 2 × 20 runs were carried out. LCST measurement was repeated three times for each sample. It should be mentioned that DLS analysis was also used at a temperature of 25 °C to measure particles’ size and zeta potential. All data were averaged over three measurements.

#### SEM and TEM imaging

To estimate the morphology of fabricated NG systems, transmission electron microscopy (TEM) and scanning electron microscopy (SEM) imaging were used. Samples were prepared by three basic steps including sonication (15 min), filtration (Millipore filter, 0.5 mm) and dilution (50–100 μg/mL), respectively^70^. In SEM analysis, prepared samples were placed on a carbon-coated slip sputtered with a thin layer of gold, and analyzed using a SEM microscope (Hitachi s 4160, Netherlands). For TEM analysis, the NG samples were placed onto carbon-coated copper grids. The micrographs were obtained using a TEM microscope (Philips cm300, Japan) with a voltage of 200 kV.

#### Drug loading (DL) and encapsulation efficiency (EE)

Curcumin (CUR), poorly water-soluble drug, was used as a drug agent. DL experiments of NG carriers were done as a following procedure. Briefly, 5 mg/mL of NG powder was dissolved in deionized water and mixed with 0.05, 0.125, 0.25, 0.375, 0.5, 0.75, 1.25, 1.75, 2.5 and 5 mg/mL of CUR to obtain NG/CUR ratio of 1:100, 2.5:100, 5:100, 7.5:100, 10:100, 15:100, 25:100, 35:100, 50:100 and 100:100 respectively. The final volume of each solution was exactly 5 mL which was incubated at controlled condition (20 °C, 250 rpm stirring, 24 h). The drug-loaded NG systems prepared by above-mentioned procedure were filtered with amicon ultra centrifugal filter (molecular weight cutoff of 10 kDa) through the centrifugation (7000 rpm, 30 min, room temperature). Concentration of the CUR in the supernatant was recorded through the measuring its absorption by UV–visible spectrophotometer at 425 nm. Then, DL capacity and EE of NG systems were determined via the following equations^7^.

$${\text{DL}} = \left( {\frac{{{\text{drug}}\; {\text{content}}\; {\text{in}}\; {\text{nanogel}}}}{{{\text{drug - loaded }}\;{\text{nanogel}}\; {\text{content}}}}} \right) \times 100$$

$${\text{EE}} = \left( {\frac{{{\text{drug}}\; {\text{content}}\;{\text{ in}} \;{\text{nanogel}}}}{{{\text{total }}\;{\text{content}} \;{\text{of}}\; {\text{primary}}\; {\text{drug}}}}} \right) \times 100$$

It is noteworthy that for DL and EE analyses, calibration curve was generated before real sample measurement. Actually, it has a critical role to adjust measurements taken on samples with unknown values (such as NG systems). Calibration curve was generated with serial dilutions of the CUR stock solution. Data were corrected by subtracting the corresponding blank absorbance. Calibration curve determination has been shown in detail in Fig. S3 (supporting information).

#### Drug release process

The CUR release from the NG systems was investigated in defined conditions such as temperatures, pH and thirteen distinctive time intervals. Three independent temperature treatments were applied as follows: (1) temperature treatment at two degrees Celsius lower than the LCST of NG, (2) that at two degrees Celsius above the LCST of NG and (3) temperature of 10 °C (contractually, too far from LCST). Treatments of pH value refer to sources of PBS buffer with pH values of 6.2 and 7.4. Actually, these six treatments (three different temperatures with two different pH values) were applied to evaluate drug release profile of each NG system. In brief, drug-loaded NG samples (1 mg/mL) were dissolved in deionized water, transferred to dialysis membrane, immersed fully into 25 mL of PBS and placed at shaker in incubator (stirred at 50 rpm) under controlled conditions. For scheduled time intervals (0.5, 1, 2, 4, 7, 10, 16, 24, 36, 48, 54, 62 and 72 h), 1 mL of the released-drug sample was withdrawn and refilled with an equal volume of fresh PBS buffer (to keep the releasing volume constant). The amount of drug released over times in all collected samples were monitored at 425 nm. In the end, the recorded values were analyzed through the CUR’s standard calibration curve.

#### MTT assay

In order to evaluate cell viability and cytotoxicity induced by NG systems, MTT assay was used. To do this, hella cell line (prepared from Pasteur Institute Iran cell bank, Tehran, Iran) were seeded (1 × 10^4^) into 96-well plates for 24 h. Drug-loaded and drug-free NG systems were used by concentrations of 10, 20, 40, 80 and 160 µM as treatments of this assay. In addition, free CUR was applied as the positive control. Prepared concentration serial was treated on three distinctive 96-well plates which contain hella cells and incubated individually for period times of 24, 48 and 72 h under optimum growth condition. MTT solution (20 mL, 5 mg/mL) was added to each well and incubated at 37 °C for 4 h. Data was recorded using a micro plate reader (ELx800, Biotek, USA) at 570 nm.

## Conclusion

Similar reports are published every year and pushed the biomedical finding toward the new aspects of diagnosing, caring and healing. In this research, the LNDNG system with a novelty in the structure and function was designed and synthesized. This nanogel was successfully prepared via grafting synthetic copolymer of P(NIPAM-co-DMAEMA) from bio-based lignin polymer as macroinitiator through “grafting from” ATRP method. The ability of LNDNG CUR drug delivery was evaluated in. The obtained results indicated that the LNDNG system has excellent features in response to stimuli effects (both temperature and pH). Proper response to stimuli, in combination with high drug loading capacity, efficient encapsulation and sustained release of drug are the main findings in the present work. High tendency of the LNDNG system in absorption of the CUR and its sustained release can be attributed to the presence of lignin. High surface area to-volume ratio arising from nanometric scale of lignin structure guaranteed maximum rate of drug loading while cross-linked P(NIPAM-co-DMAEMA) copolymer protected the whole system from undesired disruptions. Moreover, cell toxicity of the LNDNG system was found to be at the minimum level and could be ignored. Remarkable LCST behavior at the pH range of 6–8 make the LNDNG system as an appropriate vehicle for drugs delivery application.

## Supplementary Information

Supplementary Information.
