# Supplementary material for: Design and fabrication of dual responsive lignin-based nanogel via “grafting from” atom transfer radical polymerization for curcumin loading and release
Source: Sci Rep. 2021 Jan 21;11:1962. doi: 10.1038/s41598-021-81393-3 (PMC7820611; doi:10.1038/s41598-021-81393-3)
Supplement: Supplementary file 1 — Supplementary Information. [file 41598_2021_81393_MOESM1_ESM.docx]

**Design and Fabrication of Dual Responsive Lignin- Based NG via “Grafting from” Atom Transfer Radical Polymerization for Curcumin Loading and Release**

Ali Dinari^1^, Mahdi Abdollahi^1^*, Majid Sadeghizadeh^2^

1-Polymer Reaction Engineering Department, Faculty of Chemical Engineering, Tarbiat Modares University, Tehran, Iran.

2- Department of Genetics, Faculty of Biological Sciences, Tarbiat Modares University, Tehran, Iran

* Corresponding authors: Mahdi Abdollahi, Associate Professor of Polymer Reaction Engineering

Address: Faculty of Chemical Engineering, Tarbiat Modares University

Email: [abdollahim@modares.ac.ir](mailto:abdollahim@modares.ac.ir) Phone: (+9821) 82884959

**Characterization of lignin macroinitator**

50 mg of brominated lignin was dissolved in tetrahydrofuran (THF) and located at room temperature for 30 minutes. The molecular weight and its distribution of macroinitator were determined by gel permeation chromatography (GPC). Device model and applied column were Agilent-1100 series and PL gel mixd C-10 μm respectively. The obtained data are shown in table S1.

Table S1: Results of GPC analysis of brominated lignin macroinitator by GPC

| Temperature (^o^C) | Eluent | M_w_ (g/mol) | M_n_ (g/mol) |
| --- | --- | --- | --- |
| 30 | **THF** | **3501** | **2146** |

**Characterization of L-g-PNIPAM and L-g-PDMAEMA via FT-IR and ^1^H NMR**

Structure characterization of two synthesized homopolymers including L-g-PNIPAM and L-g-PDMAEMA were analyzed through the Fourier-transform infrared spectroscopy (FT-IR) and proton nuclear magnetic resonance spectroscopy (^1^HNMR). To do this, 20 mg of each sample powder was used. FT-IR analysis was recorded by PerkinElmer device (Frontier, USA). Deuterated DMSO (DMSO-d_6_) was used as the solvent in ^1^H NMR analysis. The ^1^H NMR spectra were recorded by Bruker 400 MHz spectrometer (UltraShield, Germany).

­­

**1155 cm^-1^**

Figure S1. FT-IR spectra of L-g-PNIPAM (the lower one) and L-g-PDMAEMA (the upper one)


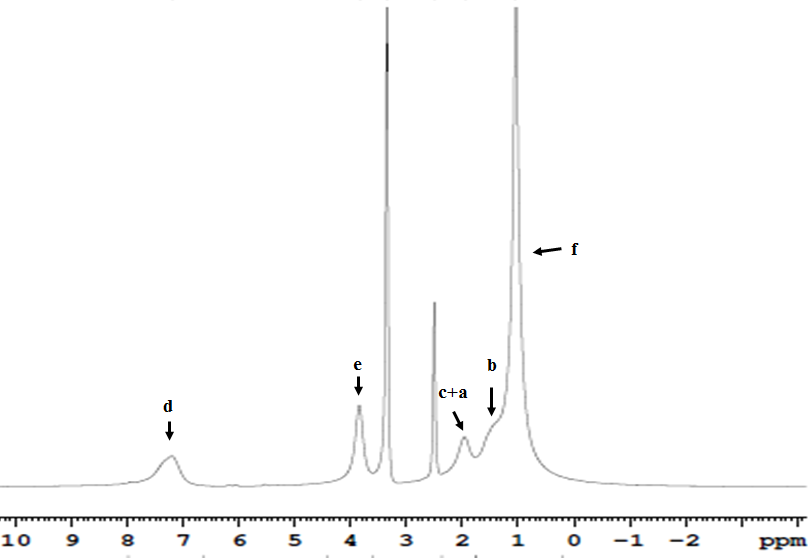


Figure S2: ^1^H NMR spectrum of L-g-PNIPAM recorded in DMSO-d_6_. The main functional groups are as follows: d) lignin aromatic rings, e) methoxyl group, c+a) bromine (Br) groups, b) methylene group of NIPAM and f) methyl group of NIPAM

**DLS analysis**

Details of NG systems including particle size, surface charges and dispersion index were determined by DLS (Table S2). In this respect, drug- free NGs refer to those samples which do not have CUR in their own structures. Acidic samples refer to those NGs which prepared at acidic condition (pH under 6). Basic ones refer to NGs which prepared at basic condition (pH above 8.). Drug- loaded samples refer to those loaded with CUR. NGs of L-g-PNIPAM and L-g-PDMAEMA do not loaded with CUR.

Table S2. DLS results of various NG systems

| Part A | | | | | | | | | | | | | | | | | | | | |
| --- | --- | --- | --- | --- | --- | --- | --- | --- | --- | --- | --- | --- | --- | --- | --- | --- | --- | --- | --- | --- |
|  | **LNDNG 1 (Copolymer I)** | | | | **LNDNG 2 (Copolymer II)** | | | | **LNDNG 3 (Copolymer III)** | | | | **LNDNG 4 (Copolymer IV)** | | | | **Reference sample** | | | |
|  | **Drug- free** | | **Drug loaded** | | **Drug- free** | | **Drug loaded** | | **Drug- free** | | **Drug loaded** | | **Drug- free** | | **Drug loaded** | | **Drug- free** | | **Drug loaded** | |
|  | **acidic** | **basic** | **acidic** | **basic** | **acidic** | **Basic** | **acidic** | **basic** | **acidic** | **basic** | **acidic** | **basic** | **acidic** | **basic** | **acidic** | **basic** | **acidic** | **basic** | **acidic** | **basic** |
| Size | **168.6** | **156.3** | **205.1** | **118.4** | **225.9** | **193.4** | **250.3** | **201.1** | **195.3** | **183.9** | **171.9** | **155.4** | **319.5** | **262.5** | **250.5** | **167.8** | **296.1** | **114.4** | **306.1** | **154.1** |
| PDI | **0.26** | **0.25** | **0.14** | **0.13** | **0.28** | **0.17** | **0.18** | **0.18** | **0.32** | **0.34** | **0.21** | **0.34** | **0.23** | **0.27** | **0.94** | **0.93** | **0.31** | **0.22** | **0.11** | **0.41** |
| zeta | **-5.5** | **-11.5** | **-12.4** | **-20.9** | **-4.8** | **-13.7** | **-20.7** | **-32.1** | **-5.9** | **-9.8** | **-12.9** | **-11.3** | **-10.3** | **-10.9** | **-21.3** | **-22.5** | **26.8** | **25.7** | **6.5** | **2.7** |
| Part B | | | | | | | | | | | | | | | | | | | | |
|  | **NG contain L-g-PNIPAM** | | | | | | | | | | **NG contain L-g-PDMAEMA** | | | | | | | | | |
|  | **Asidic** | | | | | **basic** | | | | | **Asidic** | | | | | **basic** | | | | |
| Size | **176.0** | | | | | **131.3** | | | | | **269.35** | | | | | **435.9** | | | | |
| PDI | **0.14** | | | | | **0.11** | | | | | **0.34** | | | | | **0.50** | | | | |
| zeta | **10.9** | | | | | **7.6** | | | | | **15.2** | | | | | **13.1** | | | | |

**Calibration curve:**

In order to investige drug loading and drug release processes by each NG, a calibration curve is necessary. To do this, serial dilutions of the stock solution were prepared and analyzed by UV-Visible at wavelength of 425 nm (figure S3). These recorded data were used to determine CUR concentrations in unknown samples.

Figure S3: Calibration curve for CUR drug

**Drug release:**

Release process of CUR from NG systems represent different behavior in dealing with temperature. Figure S4 (a and b) shows drug release profile of same NG (LNDNG3) in responds to LCST point (temperature 37 °C) and basic temperature (10 °C) respectively. Figure S5 shows release profile of two NG (LNDNG1 and reference sample) in response to the same temperature (temperature of 32 °C) and the same LCST.


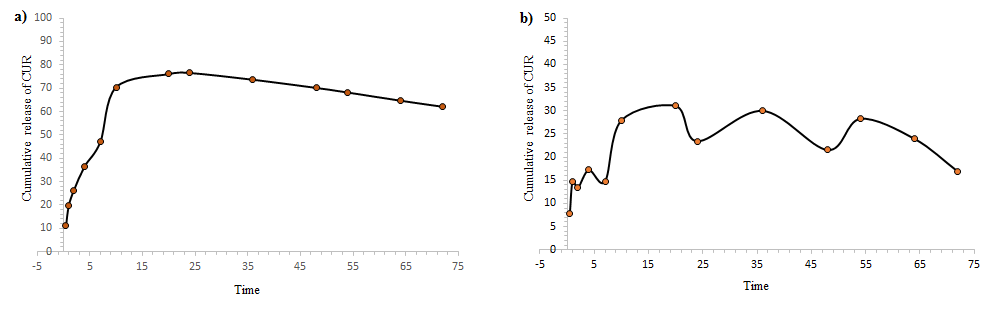


Figure S4: the CUR release profile monitoring of same NG (LNDNG3) at two different temperatures. a) Release profile at temperature of 37^˚c^ with apparent LCST occurrence and its effect on release profile. b) Release profile at temperature of 10 °c, which LCST occurrence not involved. Parameter of pH at two experiment was the same (pH= 7.4)

Figures S5: Comparison of CUR release profile at two different pH values of 7, 4 and 6.2
